# Patatin-related phospholipase A, pPLAIIIα, modulates the longitudinal growth of vegetative tissues and seeds in rice

**DOI:** 10.1093/jxb/erv402

**Published:** 2015-08-18

**Authors:** Guangmeng Liu, Ke Zhang, Jun Ai, Xianjun Deng, Yueyun Hong, Xuemin wang

**Affiliations:** ^1^National Key Laboratory of Crop Genetic Improvement, College of Life Sciences and Technology, Huazhong Agricultural University, Wuhan 430070, China; ^2^Department of Biology, University of Missouri, St. Louis, MO 63121, USA; ^3^Donald Danforth Plant Science Center, St. Louis, MO 63132, USA

**Keywords:** Cellulose, dwarf, gibberellin, longitudinal growth, phospholipase A, phospholipids, rice seeds.

## Abstract

Patatin-related phospholipase A (pPLA) hydrolyses glycerolipids to produce fatty acids and lysoglycerolipids. The *Oryza sativa* genome has 21 putative pPLAs that are grouped into five subfamilies. Overexpression of *OspPLAIIIα* resulted in a dwarf phenotype with decreased length of rice stems, roots, leaves, seeds, panicles, and seeds, whereas *OspPLAIIIα*-knockout plants had longer panicles and seeds. *OspPLAIIIα*-overexpressing plants were less sensitive than wild-type and knockout plants to gibberellin-promoted seedling elongation. *OspPLAIIIα* overexpression and knockout had an opposite effect on the expression of the growth repressor *SLENDER1* in the gibberellin signalling process. *OspPLAIIIα*-overexpressing plants had decreased mechanical strength and cellulose content, but exhibited increases in the expression of several cellulose synthase genes. These results indicate that *OspPLAIIIα* plays a role in rice vegetative and reproductive growth and that the constitutive, high activity of OspPLAIIIα suppresses cell elongation. The decreased gibberellin response in overexpressing plants is probably a result of the decreased ability to make cellulose for anisotropic cell expansion.

## Highlight

The phospholipase pPLAIIIα plays an important role in rice vegetative and reproductive growth, and constitutive, high expression of *OspPLAIIIα* suppresses cellulose production and cell elongation.

## Introduction

Patatin-related phospholipase A (pPLA) is a major family of plant lipid acyl hydrolases that hydrolyse the acyl ester bond at either the *sn*-1 or *sn*-2 position of glycerolipids to produce free fatty acid and lysophospholipids. Recent studies in *Arabidopsis* have indicated that pPLAs are involved in various plant processes, including response to abiotic and biotic stresses, hormone signalling, growth, and development (Wang, 2004; La Camera *et al.*, 2005; Yang *et al.*, 2007, 2011; Rietz *et al.*, 2010; Scherer *et al.*, 2010; Li *et al.*, 2011). The 10 *Arabidopsis* pPLAs are grouped into three subfamilies, pPLAI, pPLAII (α, β, γ, δ, and ε), and pPLAIII (α, β, γ, and δ). pPLAI has the highest homology to animal calcium-independent iPLA_2_ (Winstead *et al.*, 2000; Holk *et al.*, 2002) and is implicated in plant response to pathogen infection (Yang *et al.*, 2007) and auxin (Effendi *et al.*, 2014). AtpPLAIIα negatively regulates the production of oxylipins, such as jasmonates, and plays a role in cell death (La Camera *et al.*, 2009) and drought tolerance (La Camera *et al.*, 2005; Yang *et al.*, 2012). AtpPLAIIβ has been implicated in plants coping with phosphate deficiency, and pPLAIIγ, δ, and ε in plant response to hormone treatment and nutrient starvation (Rietz et al., 2010).

Compared with pPLAI and pPLAIIs, pPLAIIIs lack the canonical S–D dyad esterase motif in their catalytic centres, the serine (S) residue in the GxSxG esterase box is replaced by glycine (G) and the aspartate (D) residue in the conserved DGG motif is also replaced by G (Li *et al.*, 2011). Despite such changes, pPLAIIIs have been shown recently to have acyl-hydrolysing activity (Li *et al.*, 2011, 2013). In *Arabidopsis*, an activation-tagged mutant of *pPLAIIIδ*, *STURDY*, exhibited stiff inflorescence stems, thicker leaves, shorter siliques, and round-shaped flowers (Huang *et al.*, 2001). Similarly, overexpression of *AtpPLAIIIβ* resulted in shorter leaves, hypocotyls, petioles, and primary roots (Li *et al.*, 2011). Further analysis revealed that altered *AtpPLAIIIβ* expression affected cellulose content (Li *et al.*, 2011). Recent results indicate that *pPLAIIIδ* participates in plant response to auxin (Labusch *et al.*, 2013), and overexpression of *pPLAIIIδ* also leads to improved oil content of seeds and the ratio of long chain fatty acids (Li *et al.*, 2013).

In comparison, little is known about the function of *pPLA* genes in crop plants. Overexpression of an *Oncidium* pPLA *OSAG78*, closely related to *Arabidopsis pPLAIIIδ*, delayed *Arabidopsis* flowering by reducing gibberellin synthesis, as well as shortening the length of flowers, stamens, and pistils when compared with the wild type (WT) (Lin *et al.*, 2011). The rice mutant *DEP3* (*DENSE and ERECT PANICLE 3*), which was mapped in the *OspPLAIIIδ* gene, was isolated in a screen for alterations in panicle morphology (Qiao *et al.*, 2011). Panicle architecture is an important agronomic and yield trait for cereal crops, and the rice genome contains six *pPLAIII* genes (*α*, *β*, *γ*, *δ*, *ε*, *ζ*). Thus, this study was undertaken to determine the function of *pPLAIII* genes in rice growth and development. The initial focus was on *OspPLAIIIα* because the function of OspPLAIIIα was unknown in rice and *Arabidopsis*. In addition, microarray and PCR analyses show that the transcript level of rice *OspPLAIIIα* increased under salt and drought conditions (Amarjeet *et al.*, 2012). A gene knockout (KO) mutant was isolated and *OspPLAIIIα*-overexpressing (OE) rice plants were also produced, and the effect of the genetic manipulations of *OspPLAIIIα* on rice were analysed.

## Materials and methods

### Plant materials and growth condition

*Oryza sativa* spp. Japonica cultivar Dongjin was used as the WT for reverse transcription–PCR (RT–PCR) gene expression studies and for overexpression transformation. A mutant line of *OspPLAIIIα* (Os03g14950) was identified from a *Dissociation* (*Ds*) insertion transposon population derived from Dongjin and obtained from the Rice Division, Yeongnam Agricultural Research Institute, National Institute of Crop Science, Milyang, Korea. The insertion mutation was confirmed by PCR, and homozygous mutant plants were isolated following the selection of antibiotic resistance and PCR verification. Plants were grown in the field, greenhouse, or liquid medium. For gibberellin (GA; Sigma-Aldrich) treatment experiments, five-leaf stage WT, *OspPLAIIIα*-KO, and *OspPLAIIIα*-OE seedlings were sprayed with 500 μM GA_3_ for 13 d, once every 3 d, then leaves of the control and treated seedlings were sampled for observation and RNA extraction.

### Vector construction and plant transformation

The gene region of *OspPLAIIIα* in the rice genome is 1407bp long, including two exons and one intron. The primer sequences used for overexpression vector construction are 5′-GGGGTACCCCGGGTTTTGGCAATTGGCATGG-3′ and 5′-CGCGGATCCGCGGCCCATCGCCGCCACCGCCGC-3′, including *Kpn*I and *Bam*HI restriction sites, respectively. The gene was amplified by PCR (95 °C for 40 s, 58 °C for 40 s, and 72 °C for 2min). The fragment was cloned into the pCAMBIA1301U vector with a Flag-tag fused to the C-terminus. The expression of *OspPLAIIIα* was under the control of the maize ubiquitin promoter. The construct was transformed into rice callus by *Agrobacterium tumefaciens*-mediated transformation (Lin and Zhang, 2005).

### RNA extraction and quantitative real-time PCR

RNA was extracted from liquid nitrogen-frozen plant leaves using TransZol reagent according to the manufacturer’s instruction (TransGen; http://www.transgen.com.cn). DNase I was applied to remove DNA, and DNA-free RNA was used for the first-strand cDNA reverse transcription synthesis using a TIANscript RT Kit (Tiangen). Primer sequences for specific genes are listed Supplementary Table S1 available at *JXB* online. Quantitative real-time PCR was performed with the TransStart Tip Green Supermix (TransGen). Amplification of rice *β-actin* was used as a reference. The PCR conditions were: 95 °C for 30 s, 55 °C for 30 s, and 72 °C for 30 s for 55 cycles. Two rice genes, *β-actin* and *ubiquitin 5* (*UBQ5*), were used as internal controls, and both of them were expressed at a similar level among WT, KO, and OE plants (Supplementary Fig. S1 at *JXB* online). The level of transcripts was normalized to *β-actin*.

### Breaking force measurements

Breaking force is defined as the weight (g) needed to break the stem. For measurement of the force, a hoop tied to a container was placed in the middle of a stem. Water was added to the container until the stem broke. Multiple stems from each genotype were tested, and the results were analysed using Student’s *t*-test.

### Protein extraction and immunoblotting

The proteins of rice leaves were extracted at 4 °C in pre-cooled buffer B [50mM Tris–HCl pH 7.5, 150mM NaCl, 50mM sucrose, 1mM phenylmethylsulphonyl fluoride (PMSF), 0.1% Triton X-100], containing one tablet/50ml of protease inhibitor mixture (Roche, Roche Applied Science, Mannheim, Germany). The homogenates were centrifuged at 10 000 *g* for 20min at 4 °C, and the supernatant was used for immunoblotting. Total proteins were separated by 8% SDS–PAGE and then transferred to a polyvinyldifluoridene (PVDF) membrane. The membrane was pre-blotted with 5% non-fat milk, incubated with Flag/GFP (green fluorescent protein) primary antibodies for 2h, and then incubated with the secondary IgG–alkaline phosphatase antibodies for 2h at room temperature. After washing with phosphate-buffered saline–Tween-20 (PBST) three times, the membrane was incubated with an alkaline phosphatase substrate containing NBT [30mg nitroblue tetrazolium+70% dimethylformamide (DMF) ml^–1^], 5-bromo-4-chloro-3-indolyl phosphate (15mg BCIP+1ml of 15mg ml^–1^ DMF), and Tris buffer (2.1g l^–1^ Tris+0.12g l^–1^ MgCl·H_2_O), (1:1:100, v/v/v) at room temperature for colour development.

### Histological analysis

For microscopic observation, young leaves, roots, and leaf sheaths were fixed in ethanol:glacial acetic acid (9:1, v/v) for 12h at room temperature and then transferred to a solution containing chloral hydrate, glycerol, and H_2_O (8:1:2, w/v/v) with gentle shaking for 24h. Samples or sample sections were then observed under a microscope.

### Plant cell wall fractionation and cellulose content measurements

Cellulose and hemicelluloses were extracted using the plant cell wall fractionation method (Peng *et al.*, 2000). After pectin were removed from the crude cell wall fraction using ammonium oxalate [0.5% (w/v)] in a boiling water bath for 1h, the hemicelluloses was extracted from the remaining pellet with a solution composed of 4M KOH and 1.0mg ml^–1^ sodium borohydride at 25 °C for 1h. The residue was further extracted with acetic acid, nitric acid, and water (8:1:2, v/v) to separate the remaining hemicelluloses, heating for 1h at 100 °C, and the remaining pellet was cellulose. Anthrone–sulphuric acid was used to measure the cellulose content (Peng *et al.*, 2000).

### Lipid extraction and analysis by mass spectrometry

Lipids were extracted from fully expanded leaves and analysed by electrospray ionization–tandem mass spectrometry (ESI-MS/MS), and the levels of the molecular species phosphatidic acid (PA), phosphatidylcholine (PC), phosphatidylethanolamine (PE), phosphatidylglycerol (PG), phosphatidylinositol (PI), phosphatidylserine (PS), monogalactosyldiacylglycerol (MGDG), and digalactosyldiacylglycerol (DGDG) were determined as previously described (Welti *et al*., 2002) with modifications described by Xiao *et al.* (2010). Briefly, three replicates of fully expanded leaves at the tillering stage were excised and immediately immersed in isopropanol containing 0.01% butylated hydroxytoluene at 75 °C followed by four rounds of chloroform and methanol extraction. Extracts for each replicate were combined and washed with 1M KCl. After extraction, delipidated tissue was oven dried at 100 °C overnight and weighed. The phospholipids and galactolipids were quantified in comparison with phospholipid and galactolipid internal standards.

## Results

### The *OspPLA* gene family in rice

**Fig. 1.**
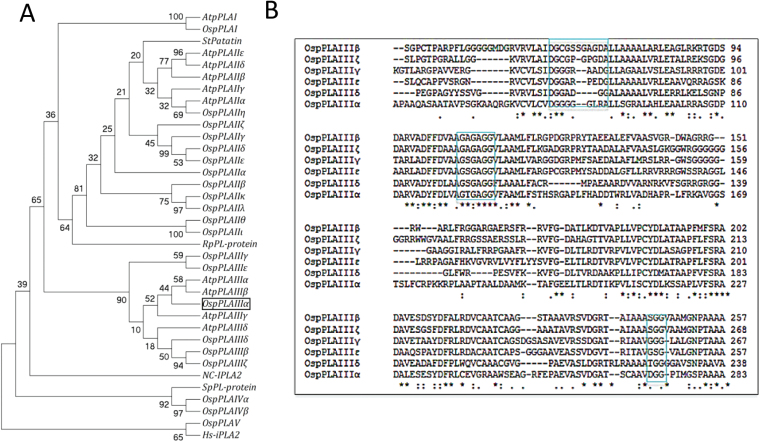
Phylogenic relationship of pPLAs in *Oryza sativa* and other species, and sequence alignments. (A) The cladogram of putative pPLAs from *Oryza sativa* and their relationship with pPLAs from other species based on protein sequences was produced with MEGA4. AtPLAI, IIα, IIIα, *Arabidopsis* patatin-like acyl hydrolase I, IIα, and IIIα (At1g61850, At2g26560, At2g39220, *Arabidopsis thaliana*); StPatatin, a potato class I patatin precursor (P11768, *Solanum tuberosum*); Hs-iPLA2, human intracellular membrane-associated calcium-independent phospholipase A2 (EAL24384, *Homo sapiens*); RpPL-protein, a patatinB1 precursor from the bacterium *Rickettsia prowazekii* (CAA15046); SpPL-protein, a putative patatin-like protein from fission yeast (CAB16355, *Schizosaccharomyces pombe*); Nc-iPLA2, an iPLA2-related protein from *Neurospora crassa* (CAE76294); the nomenclature of OsPLA is shown in Supplementary Table S1 at *JXB* online. (B) Alignment of amino acid sequences of rice pPLAIII subfamilies with Clustal W2. The conserved motifs are marked with boxes; the phosphate- or anion-binding element is DGGGx(xx)G, the esterase box is GxGxG but not GxSxG, and the catalytic dyad-containing motif is GGG, SGG, TGG, TGG, or DGG.

Based on the amino acid sequence similarity to the 10 *Arabidopsis* pPLAs and the blast result of PF01734, the ID number of potato tuber patatin protein, 21 putative pPLAs were identified and named in *Oryza sativa* (Fig. 1A; Supplementary Table S2 at *JXB* online). These OspPLAs were classified into five subfamilies according to the amino acid sequence similarity to the three AtpPLAs subfamilies, and were named as OspPLAI, OspPLAII (α, β, γ, δ, ε, ζ, η, θ, ι, κ, λ), OspPLAIII (α, β, γ, δ, ε, ζ), OspPLAIV (α, β), and OspPLAV (Fig. 1A).

Similar to the *Arabidopsis* pPLAIII subfamily, the serine (S) residue in the esterase box GxSxG in OspPLAIIIs were replaced with glycine (G). The aspartate (D) residue in the conserved DGG motif was replaced with G, Thr (T), or S (Fig. 1B). pPLAIIIα is the only member of the OspPLAIII subfamily possessing a D residue in the conserved DGG motif (Fig. 1B). *OspPLAIIIα* encodes a protein of 469 amino acids with the predicted pI of 6.21 and mol. wt of 49.7kDa (Fig. 1B). The function of *OspPLAIIIα* was analysed further because the function of *OspPLAIIIα* was unknown in rice or *Arabidopsis*.

### Knockout and overexpression of *OspPLAIIIα* in rice

**Fig. 2.**
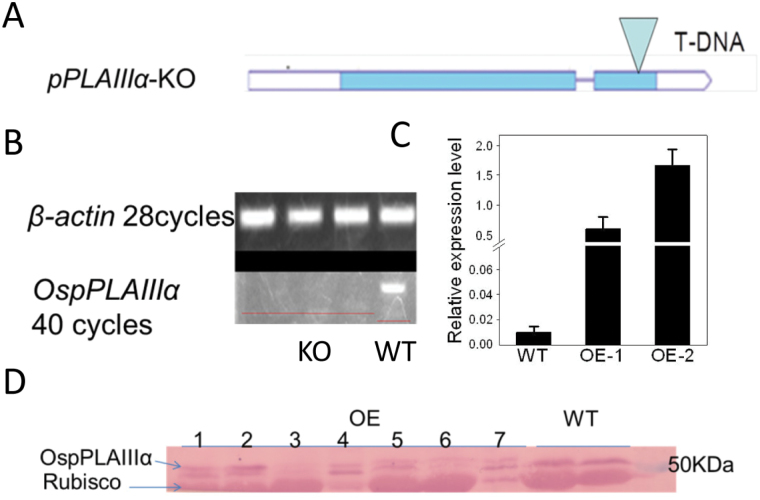
*OspPLAIIIα* T-DNA insertion mutant and overexpression in rice. (A) Schematic map showing the T-DNA insertion location (PEG_3A-11040.R) in *OspPLAIIIα.* (B) PCR verification of the *OspPLAIIIα* transcript level in the mutant. RNA was extracted from the same stage rice leaves from mutant and wild-type seedlings grown in a greenhouse. The RNA level was normalized to that of *β-actin*. (C) Transcript level of *OspPLAIIIα* in wild-type and overexpressing leaves at the tillering stage. The transcript level was measured by real-time PCR and normalized to the level of β-actin. Values are means ±SD (*n*=3). (D) Immunoblotting of pPLAIIIα in overexpressing and wild-type rice leaves. pPLAIIIα was fused to a Flag tag and, after SDS–PAGE separation, protein was immunoblotted with anti-Flag antibodies and visualized by alkaline phosphatase activity. The upper band was unique to OE transgenic plants.

To facilitate functional studies of *OspPLA* genes, a homozygous mutant for this gene, *OspplaIIIα-1*, was identified and isolated (Fig. 2A). No transcript of full-length *OspPLAIIIα* was detected, indicating that the mutant is a knockout (KO) (Fig. 2B). In addition, *OspPLAIIIα* was overexpressed under the control of the maize ubiquitin promoter in rice. The increased expression of *OspPLAIIIα* in *Pro*_*ubiquitin*_ *:OspPLAIIIα* (*OspPLAIIIα*-OE) was assessed using real-time PCR, and the transcript level of *OspPLAIIIα* in OE-1 and OE-2 leaves was found to be substantially higher than that of the WT (Fig. 2C). The overexpressed pPLAIIIα was fused at the C-terminus to the Flag tag. Immunoblotting using antibodies against Flag detected some non-specific bands, but the 53kDa band that was visible only in *OspPLAIIIα*-OE plants but not in the WT is the expected size of the product of the introduced *OspPLAIIIα* (Fig. 2D). The transcript and immunoblotting data together indicate that OspPLAIIIα protein is produced in the OE plants.

### Decreased height and cell elongation in *OspPLAIIIα*-OE plants

**Fig. 3.**
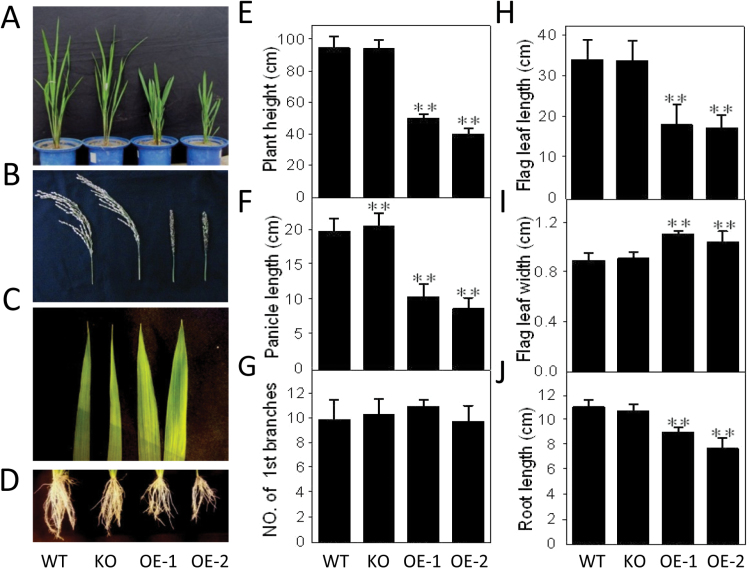
Reduced longitudinal growth in *OspPLAIIIα*-overexpressing plants in rice. (A–D) Morphology of seedlings, panicles, flag leaves, and roots from two independent *OspPLAIIIα*-overexpression lines, OE-1 and OE-2, knockout (KO), and wild type (WT) plants. Except for panicles that were collected at the maturation stage, all other tissues were sampled at the tillering stage grown in the field. (E) Plant height of wild-type, KO, and OE plants at the maturation stage. Values are means ±SD (*n*_WT_=90, *n*_KO_=90, *n*_OE-1_=4, *n*_OE-2_=4). (F and G) Length and number of first branches of the panicle. Values are means ±SD (*n*_WT_=60, *n*_HM_=60, *n*_OE-1_=28, *n*_OE-2_=28). (H and I) Flag leaf length and width at the maturation stage. Values are means ±SD (*n*=12) (J) Root length of primary roots of WT, KO, and OE plants. Values are means ±SD (*n*=12). ** indicates a significant difference compared with the wild type according to Student’s *t*-test, *P*<0.01.

The growth of *OspPLAIIIα*-OE plants was stunted (Fig. 3A). Compared with the WT, the roots, stems, leaves, roots, and panicles of OE plants were shorter (Fig. 3). At the pre-flowering stage examined, the overall plant height of OE plants was ~50% and 60% shorter, respectively, for OE-1 and OE-2 (Fig. 3E), and a similar reduction in length occurred for panicles and flag leaves (Fig. 3F, H). The width of the flag leaf increased (Fig. 3I). The leaf, root, and stem length of the *OspPLAIIIα*-KO plant was comparable with that of the WT, but its panicles were longer (Fig. 3F). Both knockout and overexpression did not affect the number of primary tillers (Fig. 3G).

**Fig. 4.**
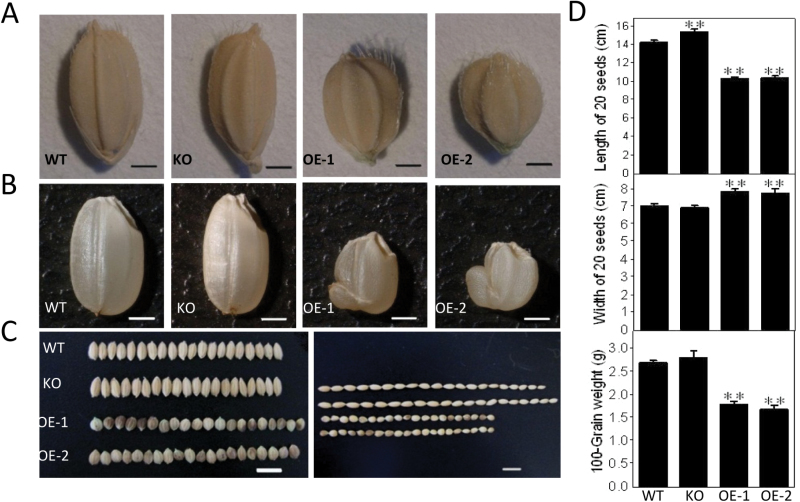
Effect of *OspPLAIIIα* alterations on seeds. (A) Seed shape of WT, KO, and OE plants. (B) Seed length and width of WT, KO, and OE plants grown in the field. (C) Quantitation of length and width of 20-grain and 100-grain weight of WT, KO, and OE seeds. Values are means ±SD (*n*=6). ** indicates a significant difference compared with the wild type according to Student’s *t*-test, *P*<0.01.

Seeds of OE lines were almost round, and were ~40% shorter and 15% wider than those of the WT (Fig. 4A, D). *OspPLAIIIα*-KO seeds were longer and the seed width remained the same as that of the WT. The 100-grain weight of *OspPLAIIIα*-OE seeds was 40% less than that of the WT (Fig. 4C, D). Endosperms of OE seeds were abnormal, with shrinkage (Fig. 4B), but OE seeds were viable.

**Fig. 5.**
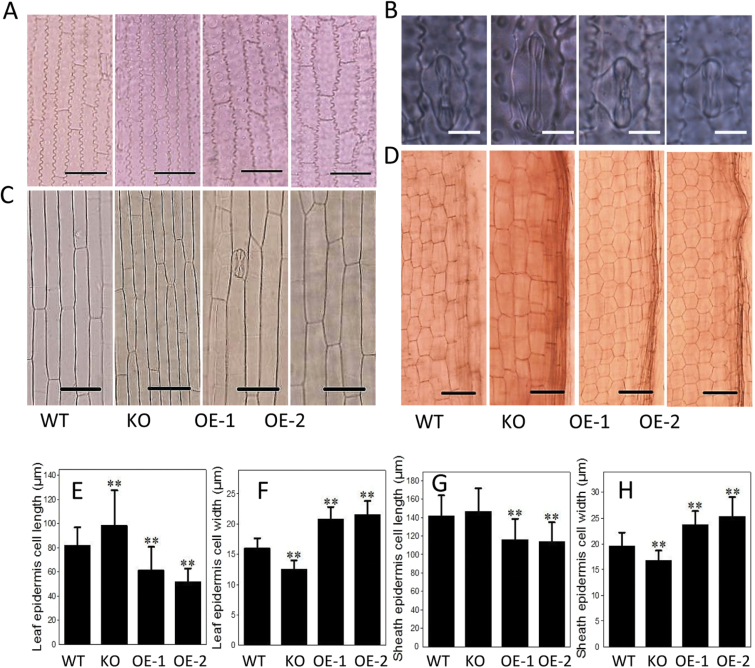
Cell size in roots of *OspPLAIIIα*-overexpressing lines and the wild type. (A) Leaf epidermis cells of WT, KO, and OE plants at the tillering stage grown in the field. Scale bar=30 μm. (B) Leaf stomata guard cells at the tillering stage. Scale bar=10 μm. (C) Sheath cells at the four-leaf stage. Scale bar=50 μm. (D) Primary root tip cells at the maturation stage. Sale bar=30 μm. (E and F) Quantitation of leaf epidermis cell length and width at the four-leaf stage. Values are means ±SD (*n*=40). (G and H) Sheath cell length and width. Values are means ±SD (*n*=50). ** indicates a significant difference compared with the wild type according to Student’s *t*-test, *P*<0.01.

When epidermis cells and guard cells of leaves at the tillering stage were compared, OE cells were shorter and wider, whereas KO cells tended to be longer and narrower than WT cells (Fig. 5A, B, E, F). Similarly, sheath cells of OE plants at the four-leaf stage were nearly 20% shorter and 20% wider, whereas KO cells were nearly 10% longer than those of the WT (Fig. 5C, G, H). When maturation zone cells of root tips were observed and measured, the cell length of *OspPLAIIIα*-OE plants was shorter than that of the WT, whereas cell width was comparable between OE and WT root cells (Fig. 5D).

### Reduced mechanical strength and cellulose content in *OspPLAIIIα*-OE plants

**Fig. 6.**
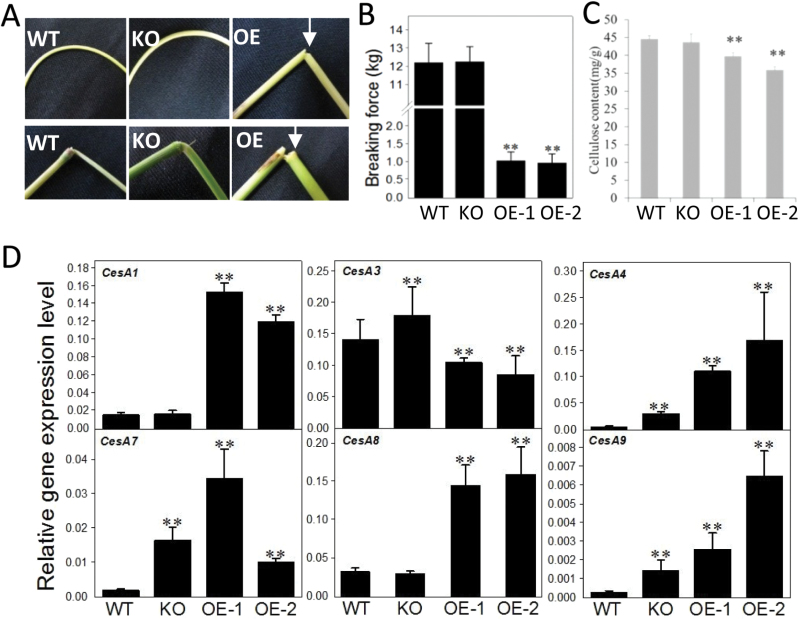
Decreased mechanical strength and cellulose content in *OspPLAIIIα*-overexpressing tissues. (A and B) Brittle stems and nodes of *OspPLAIIIα*-OE plants. (C) Breaking force of WT, KO, and OE stems. Values are means ±SD (*n*=20). (D) Cellulose content in mature leaves. Values are means ±SD (*n*=3). ** indicates a significant difference compared with the wild type according to Student’s *t*-test, *P*<0.01. (E) Transcript levels of cellulose synthase (CesA) genes of leaves at the tillering stage of plants grown in the field. The level was measured by real-time PCR and normalized to *β-actin*. Values are means ±SD (*n*=3). (This figure is available in colour at *JXB* online.)

During the handling of transgenic plants, it was noticed that the leaves and stems of OE plants were brittle. When stems were bent at a 180 ° angle, OE stems were often broken but those of of the WT were flexible (Fig. 6A). The fragility was also observed for nodes of OE plants (Fig. 6B). The mechanical strength is related to the cell wall properties, such as fibre length and strength. Cellulose is an essential component of the cell wall and plays an important role in enhanced mechanical strength (Somerville, 2006). The cellulose content was 10% lower in OE-1 and 20% lower in OE-2 than in the WT, but showed no difference between WT and KO plants (Fig. 6C).

To probe the effect on decreased cellulose content, the expression of cellulose synthase (*CesA*) genes involved in cellulose synthesis were monitored by real-time PCR using gene-specific primers (Supplementary Table S1 at *JXB* online). Compared with the WT, OE-1 and OE-2 seedlings had a higher mRNA level of *CesA1*, *CesA4*, *CesA7*, *CesA8*, and *CesA9* but a lower level of that of *CesA3* (Fig. 6E). In *OspPLAIIIα*-KO plants, the transcript level of *CesA1* and *CesA8* was comparable with that of the WT, but that of *CesA3*, *CesA4*, *CesA*7, and *CesA9* was higher than that of the WT (Fig. 6E).

### OspPLAIIIα involvement in gibberellin response

**Fig. 7.**
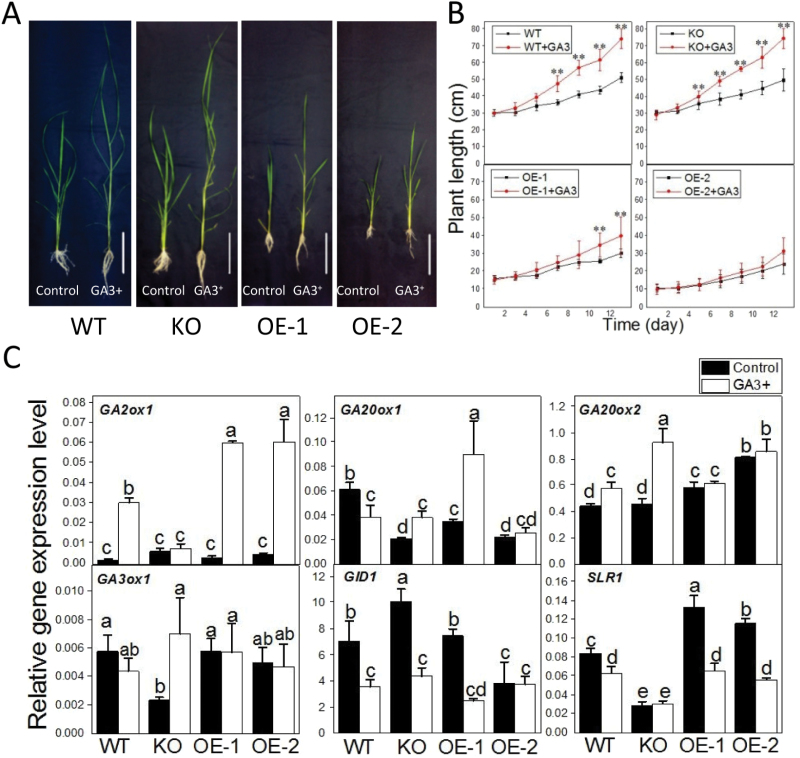
The involvement of OspPLAIIIα in gibberellin (GA) response. (A) The growth response of WT, KO, and OE seedlings grown in liquid medium without and with 500 μM GA_3_ for 14 d. (B) The plant height of *OspPLAIIIα*-alterated seedlings treated with and without GA_3_ for 14 d. Values are means ±SD (*n*=6). (C) Transcript level of genes involved in GA synthesis, degradation, and response. The level was measured by real-time PCR and normalized to *β-actin*. a, b, c, and d indicates different expression levels between treated and untreated plants within each genotypes according to ANOVA, *P*<0.05. Values are means ±SD (*n*=3). (This figure is available in colour at *JXB* online.)

GAs are plant hormones known to promote cell elongation. WT, *OspPLAIII*α-KO, and OE plants were treated with 500 μM GA_3_ for 13 d. The WT, KO, and OE plants all responded to GA_3_, and treated leaves and stems were longer and more slender than untreated controls (Fig. 7A). In the first7 d, the height of GA_3_-treated OE seedlings was not significantly different from that of untreated OE seedlings, but that of GA_3_-treated WT and KO seedlings increased nearly 30% compared with untreated seedlings. At 13 d after GA_3_ treatment, the height of WT and KO plants increased nearly 45% and 50%, respectively. In contrast, the height of OE-1 plants increased ~30% while that of OE-2 plants displayed little increase (Fig. 7B). These results could indicate that WT and KO seedlings are more responsive to the GA_3_ treatment than OE lines.

To test which GA processes are affected in the *OspPLAIIIα*-altered plants, the expression of genes involved in GA synthesis (*GA20ox1*, *GA20ox2*, and *GA3ox1*), inactivation (*GA2ox1*), and responses [*GIBBERELLIN INSENSITIVE DWARF 1* (*GID1*) and *SLENDER 1* (S*LR1*)] in the WT were compared with those of *OspPLAIII*α-KO and OE seedlings (Fig. 7C). Without GA treatment, the mRNA level of *GA20ox1* and *GA3ox1* decreased but that of *GA2ox1* increased in KO plants (Fig. 7C). In contrast, overexpression of *OspPLAIII*α did not result in much change in *GA3ox1* whereas the level of *GA20ox2* increased (Fig. 7C). *OspPLAIIIα* overexpression and knockout had an opposite effect on the expression of the growth repressor *SLR1* in the GA signalling process. Compared with that of the WT, the mRNA level of *SLR1* decreased in KO but increased in OE seedlings.

After GA_3_ treatment for 14 d, the mRNA level of *GA2ox1* in GA synthesis increased in WT and OE plants but not in KO plants (Fig. 7C). In contrast, KO plants exhibited an increase in *GA20ox2* and *GA3ox1* but no such increase occurred for the WT or OE-2. Of the two genes in GA response, the RNA level of the GA receptor *GID1* decreased in WT, KO, and OE-1 but not in OE-2 seedlings (Fig. 7C). After GA treatment, the expression of *SLR1* was down-regulated more in OE lines than in the WT, but the level of *SLR1* in the KO line was similar with or without the GA treatment (Fig. 7C).

### Changes in phospholipid content and composition in *OspPLAIIIα*-altered plants

**Fig. 8.**
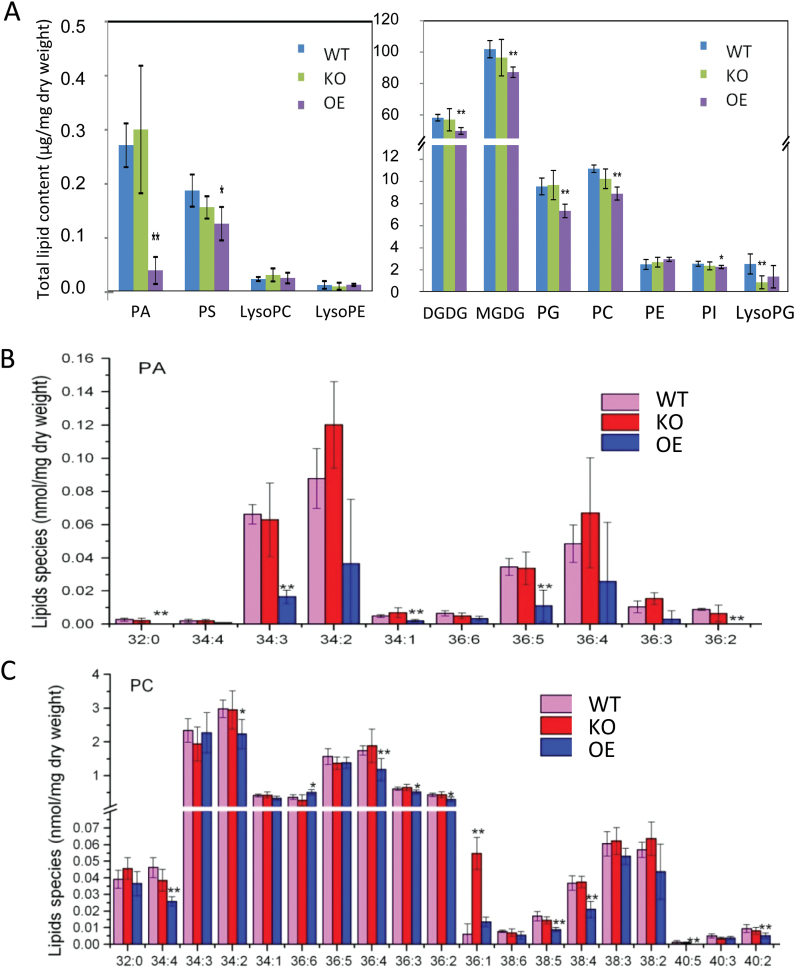
The effect of OspPLAIIIα on glycerolipid content and composition.. (A) Contents of different lipid classes from fully expanded leaves at the tillering stage of plants grown in the field. Values are means ±SD (*n*=3). (B) PA molecular species of rice leaves. Values are means ±SD (*n*=3). (C) PC molecular species of rice leaves. The numbers on the *x*-axis in (B) and (C) denote total acyl carbons:total number of acyl carbon double bonds of PA species. * and ** indicate a significant difference between OE plants and wild-type plants with *P*<0.05 and *P*<0.01 in the Student’s *t*-test. Values are means ±SD (*n*=3).

To examine the effect of *OspPLAIIIα* alterations on membrane lipids in rice, lipids from *OspPLAIIIα*-KO, OE, and WT leaves were quantitatively profiled. The content of many lipid classes in OE leaves was lower than that of the WT (Fig. 8A). In particular, the content of the minor lipid class PA in OE-1 leaves was 5-fold lower than that of WT and KO leaves (Fig. 8A, upper panel). The decrease of the major lipid classes, such as MGDG, DGDG, PC, and PG, was ~5–10% (Fig. 8A, lower panel).

Major PA species in rice are 34:2-PA, 34:3-PA, and 36:4-PA, and these major and many minor PA species were decreased in OE leaves (Fig. 8B). The decrease in PC was not as wide ranging as that of PA; six out of 19 PC species displayed some decline. The level of PC species in WT and KO leaves was comparable, except for 36:1-PC that was higher in KO than WT leaves (Fig. 8C). In comparison, there was no significant difference between KO and WT leaves in the membrane lipid classes examined except that lysoPG was lower in KO than in WT leaves (Fig. 8A, lower panel).

## Discussion

The present analysis indicates that the pPLA family in rice is much larger than that in *Arabidopsis*; the rice genome has more members of *OspPLAII* and *OspPLAIII* as well as two new subfamilies (*OspPLAIV* and *OspPLAV*). Distinguishable functions have been implicated for the three subfamilies of *Arabidopsis* pPLAI, IIs, and IIIs, and even among the individual members of pPLAIIs and pPLAIIIs (Li *et al.*, 2013). pPLAIII in *Arabidopsis* has been shown to have acyl-hydrolysing activity (Li *et al.*, 2011). Lipid analysis indicates that knockout of *OspPLAIIIα* has no apparent effect on lipid composition, but overexpression led to a large decrease in PA. This raises an intriguing question of whether PA is a substrate of OspPLAIIIα *in planta.* PA is a central intermediate in glycerolipid metabolism and also a potent mediator affecting plant growth and stress responses (Liu *et al.*, 2013). It will be of interest in future studies to determine whether the effect of OspPLAIIIα on PA content contributes to the cellular and phenotypic alteration of OspPLAIIIα-altered plants.

Stunted growth in *OspPLAIIIα*-OE plants is the most noticeable phenotype resulting from the alterations of *OspPLAIIIα* expression. OE plants were decreased in longitudinal growth in all tissues. In comparison, the effect of *OspPLAIIIα* knockout on the length of vegetative tissues was not apparent. Genetic redundancy of other *PLAIII* genes may explain the lack of overt growth alterations in vegetative tissues in KO plants. Indeed, the decreased longitudinal growth has been reported on *AtpPLAIIIδ*-OE and *AtpPLAIIIβ*-OE *Arabidopsis* and also on *OspPLAIIIδ*-OE Arabidopsis (Huang *et al.*, 2001; Li *et al.*, 2011, 2013; Lin *et al.*, 2011). These results also indicate that the function of *pPLAIII* genes in decreasing elongation is evolutionarily conserved in different species.

On the other hand, the present result showed that *OspPLAIIIα*-KO plants had longer panicles and longer seeds. A previous study reported that mutation in *OspPLAIIIδ* altered panicle morphology, and that the *pPLAIIIδ* mutant *DEP3* displayed dense and erect panicles (Qiao *et al.*, 2011). These results indicate that *OspPLAIIIα* and *pPLAIIIδ* affect rice panicle growth and development, and yet their functions differ from one another. Panicle and seed morphologies are important yield and quality traits for cereal crops. The rice genome has six *pPLAIII* genes (*α*, *β*, *γ*, *δ*, *ε*, *ζ*). The availability of the mutants will help future studies of the function of the other members of the *OspPLAIII* subfamily in combination with *OspPLAIIIα* and *pPLAIIIδ* in rice panicle architecture and seed production.

To probe the mechanism of the decreased cell and plant elongation, the plant response to the cell elongation-promoting hormone GA was tested. Leaves and stems of GA-treated WT and KO plants were longer and more slender than those of untreated controls. *OspPLAIIIα*-OE seedlings responded to the GA treatment but they were much less responsive to GA than the WT and KO plants. The expression of genes involved in GA synthesis (*GA20ox1*, *GA20ox2*, and *GA3ox1*), inactivation (*GA2ox1*), and responses (*GID1* and *SLR1*) was then compared. KO plants had decreased mRNA levels of the GA synthesis genes *GA20ox1* and *GA3ox1* but increased levels of the GA-inactivating gene *GA2ox1* without GA treatment. These changes could be a feed-back inhibition of GA responses as epidermal cells of KO plants tended to be longer than those of the WT. In GA response, the receptor GID1 binds to GA, and GID1 interacts with DELLA proteins such as SLR1 that represses GA signalling (Hirano *et al.*, 2012). SLR1 inhibits plant growth and this inhibition is suppressed by the GA-dependent GID1–SLR1 interaction. The expression of *SLR1* was down-regulated in WT and OE lines, but not in the KO after GA treatment. Without GA treatment, *OspPLAIIIα*-KO plants exhibited a decrease in the mRNA level of *SLR1* whereas OE plants had a higher level of *SLR1* than the WT. Since *SLR1* is a suppressor of cell elongation, the data are in agreement with the observation that KO plants tended to have longer epidermal cells whereas OE plants had shorter cells than the WT. After application of GA, the expression of *SLR1* was down-regulated more in OE plants than in the WT, but not in KO plants. These changes in *SLR1* expression in KO and OE plants could mean that *SLR1* is a target of *OspPLAIIIα* action. The increased *OspPLAIIIα* expression and its protein level suppress the *SLR1* transcript level in response to GA. Despite the lack of change in *SLR1* level, the KO plants responded to GA-promoted elongation similarly to the WT. The lack of suppression of elongation in KO plants could mean that besides the transcript level, another mechanism, such as post-translational modification, and interaction with lipid mediators from pPLA, such as free fatty acids and lysophospholipids, are involved in the SLR1 action to suppress the GA-promoted cell elongation.

The dwarf *OspPLAIIIα*-OE plants also showed decreased mechanical strength. The reduced mechanical strength and plant height were linked to the decrease in cellulose content of *OspPLAIIIα* plants as cellulose content plays a key role in plant mechanical strength. In addition, cellulose deposition is regarded as a driving force for cell expansion through oriented deposition of cellulose microfibrils around the cell. Therefore, the change of cellulose content may influence anisotropic cell expansion and eventually exhibit a corresponding phenotype in the whole plant. Cells in OE lines were shorter than those of the WT, a probable result of a weaker driving force due to decreased cellulose, and these phenotypes were similar to those of some cellulose-deficient mutants, such as *csi1* (*CELLULOSE SYNTHASE INTERACTIVE PROTEIN 1*) and *ctl1/pom1* (Zhong *et al.*, 2002; Gu *et al.*, 2010). Based on the above observation, it was reasoned that *pPLAIII* genes regulated longitudinal growth of plants by modulating cellulose metabolism.

*OspPLAIIIα*-KO and WT plants displayed similar plant height and cellulose content. However, leaf epidermal cells were longer and narrower in KO than in WT plants. This discrepancy between the overall plant morphology/cellulose content and the epidermal cell length could mean that the effect of *OspPLAIIIα* is specific to cell types: *OspPLAIIIα* functions primarily in the epidermal cells, but cellulose content and plant height resulted from the measurements of whole tissue comprising various cell types. On the other hand, the overexpression of *OspPLAIIIα* was under the control of a constitutive ubiquitin promoter, thus resulting in a decrease in overall plant height and cellulose content.

To explore the effect of *OspPLAIIIα* overexpression and knockout on cellulose content, the effect of *OspPLAIIIα*-OE on the level of expression of several *CesA* genes was monitored. CesAs play important roles in cellulose synthesis, using UDP-glucose to produce β-1,4-glucan (cellulose) chain polymerization (Somerville, 2006; Cantarel *et al.*, 2009). CesA proteins are arranged into multimeric protein complexes, and at least three different CesA proteins are required to form a functional complex (Taylor *et al.*, 2000; Desprez *et al.*, 2007). In *Arabidopsis*, CesA1 and CesA3 are required for cellulose biosynthesis during primary cell wall formation, whereas CesA4, CesA7, and CesA8 are needed for secondary cell wall formation (Taylor *et al.*, 2000; Persson *et al.*, 2005; Desprez *et al.*, 2007). In *OspPLAIIIα*-KO plants, the mRNA level for *CesA3*, *CesA4*, *CesA7*, and *CesA9* displayed an increase, suggesting that *OspPLAIIIα*-KO is a negative regulator of cell wall formation. On the other hand, in OE-1 and OE-2 plants, the mRNA level of *CesA3* was decreased but those of *CesA3*, *CesA4*, *CesA7*, and *CesA8* all showed an increase. The results appeared to be counterintuitive to the decreased cellulose content in OE plants. This could mean that the increase in *CesA* expression resulted from a feed-back regulation in response to decreased cellulose content.

Based on the results, it is proposed that the phenotype of decreased growth and response to GA in the overexpressors is probably a result of the decreased ability to make a cell wall, which is required for anisotropic cell expansion. The effect of *OspPLAIIIα* overexpression on the expression of genes in GA and cellulose synthesis results from decreased cellulose content and cell elongation. It is likely that the high activity of OspPLAIIIα and its lipid products perturbs cellulose production. To understand the role of *OspPLAIII* genes in plant morphology and growth, further studies are needed to determine how pPLAIIIs and associated membrane lipid changes modulate assembly and cellulose synthase activity.

## Supplementary data

Supplementary data are available at *JXB* online.

Figure S1. Relative transcript level of *UBQ5* normalized to the level of *β-actin.*

Table S1. Primers and sequences used for PCR.

Table S2. OspPLA family members

Supplementary Data

## Abbreviations:

CesA: cellulose synthase
DGDG: digalactosyldiacylglycerol
GA: gibberellin
GA2ox: GA 2-oxidase
GA3ox: GA 3-oxidase
GA20ox: GA 20-oxidase
MGDG: monogalactosyldiacyglycerol
PA: phosphatidic acid
PC: phosphatidylcholine
PE: phosphatidylethanolamine
PG: phosphatidylglycerol
PI: phosphatidylinositol
pPLA: patatin-related phosholipase A
PS: phosphatidylserine.
