# Supplementary material for: Patatin-related phospholipase A, pPLAIIIα, modulates the longitudinal growth of vegetative tissues and seeds in rice
Source: J Exp Bot. 2015 Aug 18;66(21):6945–55. doi: 10.1093/jxb/erv402 (PMC4623698; doi:10.1093/jxb/erv402)
Supplement: Supplementary Data [file supp_66_21_6945__index.html]

Patatin-related phospholipase A, pPLAIIIα, modulates the longitudinal growth of vegetative tissues and seeds in rice — Patatin-related phospholipase A, pPLAIIIα, modulates the longitudinal growth of vegetative tissues and seeds in rice — Supplementary Data 

# Patatin-related phospholipase A, pPLAIIIα, modulates the longitudinal growth of vegetative tissues and seeds in rice

## Supplementary Data

Data files

- Supplementary Data - Supplementary Data
