# Supplementary material for: Patatin-related phospholipase A, pPLAIIIα, modulates the longitudinal growth of vegetative tissues and seeds in rice
Source: J Exp Bot. 2015 Aug 18;66(21):6945–55. doi: 10.1093/jxb/erv402 (PMC4623698; doi:10.1093/jxb/erv402)
Supplement: Supplementary Data [file supp_erv402_jexbot148288_file001.pdf]

Supplemental Table 1. Primers and sequences used here.

Primer name	Sequence
Ⅲα.F	5'- GGGGTACCCCGGGTTTTGGCAATTGGCATGG
Ⅲα.R	5'- CGCGGATCCGCGGCCCATCGCCGCCACCGCCGC
For qRCR	
RT14950.F	5'- CAGACGAACGCGGAGAG
RT14950.R	5'- TGCTTGAACGCCACGGT
14950HQ.F	5'- GTGCCGCTCGTCCAACTACC
14950HQ.R	5'- GCCTGCTTGAACGCCACGGT
OsCESA1.F	5'- TTGACTTGCACGATCGATACG
OsCESA1.R	5'- TCCCACATAAACTGGACCCTG
OsCESA3.F	5'- GCATTTTTGCTACTGGCATCC
OsCESA3.R	5'- TCCCTGGAACAAAGCAAAGAG
OsCESA4.F	5'- CTAATGCGACGAAGACGATG
OsCESA4.R	5'- GATTTAACGGTGCCCTCTCA
OsCESA7.F	5'- TCCATCTTCTCCCTCGTCTG
OsCESA7.R	5'- GAATCATCCATCCGGTCATC
OsCESA8.F	5'- TGTTGAAGGTGCTGGATTCGA
OsCESA8.R	5'- TGAGAGTGGAGGCAACAAACG
OsCESA9.F	5'- TTAGCACGTTTGCGAGTTTG
OsCESA9.R	5'- GAACTCGTCGTCCTCGTCTC
GA2-OX1.F	5'- TGACGATGATGACAGCGACAA
GA2-OX1.R	5'- CCATAGGCATCGTCTGCAATT
GA20-OX1.F	5'- GCCACTACAGGGCCGACAT
GA20-OX1.R	5'- TGGTTGCAGGTGACGATGAT
GA20-OX2.F	5'- GGGAGGGTGTACCAGAAGTACTG
GA20-OX2.F	5'- GGCTCAGCTCCAGGAGTTCC
GA30-OX1.F	5'- CCATAGGCATCGTCTGCAATT
GA30-OX1.R	5'- AACTCCTCCATCACGTCACAG
GIDRT.F	5'- CAAGGTTGTCCAATGCGAGAAC
GIDRT.R	5'- AGGTTAGCGTTGAGGAAGTCGG
SLRRT.F	5'- AAGGATCGTCACCGTGGTAGAG
SLRRT.R	5'- TGGTGGAGTAGTAGTGCAGCGA
β-actin F	5'- TGCTATGTACGTCGCCATCCAG
β-actin R	5'- AATGAGTAACCACGCTCCGTCA
ubiqutin5.F	5'- CAGCAGCGGCTCATCTT
ubiqutin5.R	5'- GCTTCTTGGGCTTGGTGTA

Supplementary Table 2 OspPLAs family members

nomenclature	gene nr.	nomenclature	gene nr.
OspPLAIIα	Os01g67310	OspPLAI	Os07g33670
OspPLAIIβ	Os03g27610	OspPLAIIIα	Os03g14950
OspPLAIIγ	Os08g28880	OspPLAIIIβ	Os03g43880
OspPLAIIδ	Os08g37180	OspPLAIIIγ	Os03g57080
OspPLAIIε	Os08g37210	OspPLAIIIδ	Os06g46350
OspPLAIIζ	Os08g37250	OspPLAIIIε	Os07g05110
OspPLAIIη	Os09g28770	OspPLAIIIζ	Os12g41720
OspPLAIIθ	Os11g39990	OspPLAIVα	Os01g55650
OspPLAIIι	Os11g40009	OspPLAIVβ	Os03g59620
OspPLAIIκ	Os12g36530	OspPLAV	Os11g34370
OspPLAIIλ	Os12g36610		

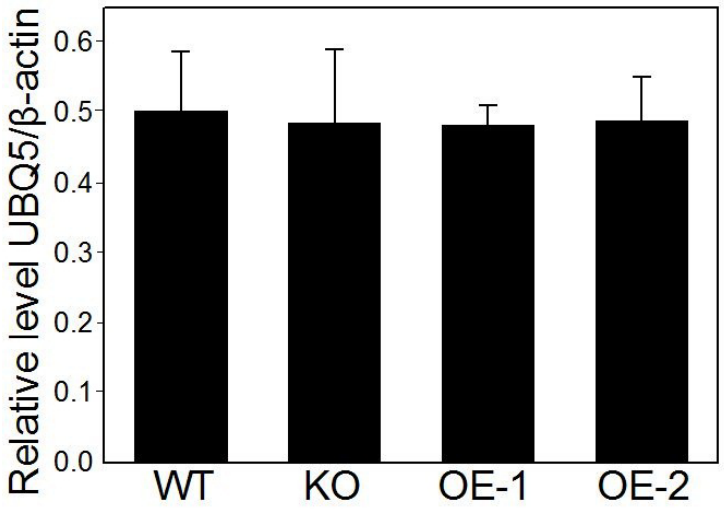

Supplemental Fig.1 . Relative transcript level of *UBQ5* normalized to the level of *β-actin.* Values are means ± SD (n = 3).
